# A theoretical model for Zika virus transmission

**DOI:** 10.1371/journal.pone.0185540

**Published:** 2017-10-04

**Authors:** Ebenezer Bonyah, Muhammad Altaf Khan, K. O. Okosun, Saeed Islam

**Affiliations:** 1 Department of Mathematics and Statistics, Kumasi Technical University, Kumasi, Ghana; 2 Department of Mathematics, City University of Science and Information Technology, Peshawar, KP, 25000, Pakistan; 3 Department of Mathematics, Vaal University of Technology, Vanderbijlpark, South Africa; 4 Department of Mathematics Abdul Wali Khan, University Mardan, KP, Pakistan; Universitatsmedizin Greifswald, GERMANY

## Abstract

In this paper, we present and analyze an SEIR Zika epidemic model. Firstly, we investigate the model with constant controls. The steady states of the model is found to be locally and globally asymptotically stable. Thereafter, we incorporate time dependent controls into the model in order to investigate the optimal effects of bednets, treatments of infective and spray of insecticides on the disease spread. Furthermore, we used Pontryagin’s Maximum Principle to determine the necessary conditions for effective control of the disease. Also, the numerical results were presented.

## 1 Introduction

Zika virus was first discovered in 1947 in Uganda among a certain Rhesus macaque population [1]. The “*Aedes*” mosquito is the vector responsible for zika virus transmission. It has also been established that there is potential transmission to humans during transplacental transmission or during child delivery from mother to child [1, 2]. There are several characteristics of Zika virus species which are associated with dengue virus and chikungunya virus [3]. Zika virus (ZIKV), a Flavivirus closely related to dengue, is primarily transmitted to humans by the bites of infected female mosquitoes from the *Aedes* genus [4]. *Aedes* mosquitos transmit Zika virus. Mosquitoes become infected by taking a blood meal from an infected person and then they pass the virus as they bite other people. There is also evidence that Zika virus can be transmitted through sex [5, 6].

The signs and symptoms of Zika virus is similar to dengue fever symptoms and the disease clinical symptoms manifest in humans within 3 to 12 days. Symptoms is often not severe and the duration is very short, which is 2-7 days, hence, it is often misdiagnosed as dengue fever. Nearly one in four persons infected by Zika virus is likely to develop the symptoms of the disease [2, 3]. Zika virus is currently spreading to many countries in South and Central Americas and the Caribbean. Precisely, anywhere “*Aedes*” species of mosquitoes be found, Zika virus infection is highly possible to can occur [7]. The spread of Zika virus across many geographical regions has attracted global attention [8].

There is presently no definite treatment for Zika virus yet, except the control of vectors using insecticide spray and destruction of the larval breeding grounds. Recent studies have revealed a devastating effect of Zika virus infection on pregnant women [7]. D. Gao et al. [4] presented a mathematical model on zika virus with prevention and optimal control.

In recent decades, mathematical modeling has played an important role in the understanding of disease epidemiology and control [9]. Lee and Pietz [10] developed a mathematical model on zika transmission with vector-host structure focusing on logistic growth in human population and dynamic growth in vector population. The authors observed that digital disease surveillance is crucial in minimizing the spread of the disease. Li et al. [11] constructed a multi-group brucellosis model comprised sheep and cattle and found out that the best way to contain the brucellosis is to avoid cross infection. They further suggested that the concept of mix feeding must be avoided. While, Sun and Zhang [12] formulated and developed a sheep brucellosis model incorporating immigration and proportional birth. The authors also took into consideration both direct and indirect transmission through animals who have been infected and the bacteria in the environment. Their study revealed that the best control strategies were the following: elimination, vaccination, reduction of migration and disinfection. In Xing et al., [13], the authors further proposed a mathematical model on H7N9 avian influenza among migrant birds, resident birds, domestic poultry and human in China. They found out that temperature cycling might be the main cause of the disease, however, controlling trading markets would help to control the spread of the disease. While Sun et al., [14] developed a cholera transmission model which focused on the disease dynamics in China. They further observed that in order to reduce the spread of the disease, immunization coverage must be improved and also the environment must be managed very well. Also, Yu and Lin [15] studied a complex dynamical behaviour in biological systems as a result of multiple limit cycles bifurcation using simple predictor-prey model. The analysis indicated that bistable phenomenon exist. A modified SIR model was developed by Gui and Zhang [16], which has a nonlinear incidence and recovery rates with the main aim was to comprehend any government intervention and hospital resources influence on diseases spread. In their studies, it was observed that the model exhibited a backward bifurcation phenomenon, which implied that reduction in the reproduction number to less than one is not sufficient enough to stop the spread of the diseases. In Li [17], a dynamical model was constructed to explain the periodic behavour of HFRS in China. He also found out that the critical issues associated with the spread of this disease is periodic transmission rates and the rodent periodic birth rate of HFRS in China.

These models are characterized with vivid qualitative accounts of the complex nonlinear process involved in the transmission process of diseases and provide insight into the dynamics of the disease. This eventually lead to proper and effective disease control strategies and management by health authorities. However, to the best of our knowledge there are very few mathematical models proposed on Zika virus.

With regard to some vector-borne diseases, such as malaria, dengue fever and Buruli ulcer, there are many mathematical models that have provided insight into the management and control of these diseases [9, 18]. For instance, Nishiura et al. [19] developed a Zika mathematical model which appeared to exhibit the same dynamics as dengue fever. While Khan et al. [20] proposed a mathematical model with saturation function to investigate the dynamic of typhoid fever. In Khan et al. [21], the authors developed a mathematical model on Leptospirosis with saturation function to explore the dynamics of the disease. The authors in Bonyah et al. [18] developed a *SIR* mathematical model to study the dynamics of Buruli ulcer and suggested that medical resources should be made available for patients in order to control the disease.

Jinhong et al. [22] constructed a *SEIR* epidemic model with saturated incidence rate and saturated treatment function and their results showed that hospital facilities should be expanded to accommodate more patients for treatment. Wan and Cui [23] constructed mathematical model to examine the impact of resources for hospitalized people and established that sufficient number of sickbeds and medical resources are as important as disease control mechanism. Shi et al. [24] proposed an HIV model with saturated reverse function to study the dynamics of infected cells. Javidi, and Nyamorady, [25] however, developed a mathematical model with a saturated function to explore the dynamics of computer virus.

In this paper, we construct a mathematical model Zika virus. The population of human is divided into four sub-lcasses, that is, *S*_*H*_, *E*_*H*_, *I*_*H*_ and *R*_*H*_ while the mosquitos population is divided into three sub-classes, namely; *S*_*V*_, *E*_*V*_ and *I*_*V*_. This paper is two fold; initially, we constrict a mathematical model and explore their mathematical results, after that an optimal control problem is formulated. Different strategies are developed for the numerical results.

The paper is arranged as follow: Section 2 presents the model description and mathematical assumptions underlying the model. In Section 3, we examine and analyze the model equilibria and stability analysis. Section 4 is constructed to obtain the mathematical results for endemic equilibrium and bifurcation analysis. The global stability for both the disease free and endemic equilibrium is presented in section 5. In section 6 a sensitivity analysis of the model is presented.

Section 7 is devoted to optimal control analysis of the model, while the numerical results are presented in Section 8 and the conclusion is presented in Section 9.

## 2 Mathematical model formulation

In this section, we take into account the human to human infection as well as the vector (mosquito) to human transmission. The model subdivide the total human population, *N*_*H*_(*t*), into susceptible humans *S*_*H*_(*t*), exposed human *E*_*H*_(*t*), infected humans *I*_*H*_(*t*), and recovered humans *R*_*H*_(*t*), so that *N*_*H*_(*t*) = *S*_*H*_ + *E*_*H*_ + *I*_*H*_ + *R*_*H*_. The entire mosquito population, denoted by *N*_*V*_(*t*), is partitioned into susceptible vector *S*_*V*_(*t*), exposed vector *E*_*V*_(*t*) and infected mosquito *I*_*V*_(*t*) and hence *N*_*V*_ = *S*_*V*_ + *E*_*V*_ + *I*_*V*_. Based on the above discursion we present the following system:

$$\begin{array}{c} \left\{ \begin{array}{c} \frac{d}{dt}S_{H}=\Lambda_{H}-\beta_{H}S_{H}\left(I_{V}+\rho I_{H}\right)-\mu_{H}S_{H},\\ \frac{d}{dt}E_{H}=\beta_{H}S_{H}\left(I_{V}+\rho I_{H}\right)-\left(\mu_{H}+\chi_{H}\right)E_{H},\\ \frac{d}{dt}I_{H}=\chi_{H}E_{H}-\left(\mu_{H}+\gamma +\eta \right)I_{H},\\ \frac{d}{dt}R_{H}=\gamma I_{H}-\mu_{H}R_{H},\\ \frac{d}{dt}S_{V}=\Lambda_{V}-\beta_{V}S_{V}I_{H}-\mu_{V}S_{V},\\ \frac{d}{dt}E_{V}=\beta_{V}S_{V}I_{H}-\left(\mu_{V}+\delta_{V}\right)E_{V},\\ \frac{d}{dt}I_{V}=\delta_{V}E_{V}-\mu_{V}I_{V}. \end{array}\right. \end{array}$$ (1)

Recruitment of susceptible humans is denoted by Λ_*H*_, while susceptible mosquito recruitment is denoted Λ_*V*_. The effective contact rate between susceptible humans and infected mosquitoes is denoted by *β*_*H*_. Here, *β*_*V*_ is the transmission rate from infected humans to susceptible vector. The effective contact rate between infected humans and susceptible humans that can result into infection is denoted by *ρ*. The disease induced mortality rate is denoted by *δ*. Natural mortality rates due to each subpopulation of human and vector compartment are denoted *μ*_*H*_ and *μ*_*V*_ respectively. *γ* and *η* are the natural and treatment rate.

Let the total dynamics of the human population given as

$$\begin{array}{c} N_{H}^{\prime }\left(t\right)=\Lambda_{H}-\mu_{H}N_{H}-\eta I_{H}. \end{array}$$ (2)

i. e.,

$$\begin{array}{c} N_{H}^{\prime }\left(t\right)+\mu_{H}N_{H}\leq \Lambda_{H}. \end{array}$$ (3)

Now integrating both sides of the above inequality and using the theory of differential inequality due to Birkhoff and Rota [26], we get

$$\begin{array}{c} 0\leq N_{H}(S_{H},E_{H},I_{H},R_{H})\leq \frac{\Lambda_{H}}{\mu_{H}}\left(1-e^{-\mu_{H}t}\right)+N_{H}\left(S_{H}\left(0\right)+E_{H}\left(0\right)+I_{H}\left(0\right)+R_{H}\left(0\right)\right)e^{-\mu_{H}t}. \end{array}$$

Now, taking, *t* → ∞, we get $0<N_{H}\leq \frac{\Lambda_{H}}{\mu_{H}}.$

The total dynamics of vector population is given by

$$\begin{array}{c} N_{V}^{\prime }\left(t\right)=\Lambda_{V}-\mu_{V}N_{V}. \end{array}$$ (4)

The exact solution of Eq (4) when *t* → ∞ is given by

$$\begin{array}{c} N_{V}\left(t\right)=\frac{\Lambda_{V}}{\mu_{V}}. \end{array}$$

It is obvious that

$$\begin{array}{ccc} \Pi & = & \{S_{H},E_{H},I_{H},R_{H},S_{V},E_{V},I_{V})\in R_{+}^{7}|0\leq S_{H}+E_{H}+I_{H}+R_{H}\leq \frac{\Lambda_{H}}{\mu_{H}}\\ & & \;and\;0\leq S_{V}+E_{V}+I_{V}\leq \frac{\Lambda_{V}}{\mu_{V}}\}, \end{array}$$

which is positively invariant, dissipative and the global attractor is attained in *Π*.

## 3 Equilibria and disease free stability

The disease free equilibrium for the model (1) is $E_{0}=\left(\frac{\Lambda_{H}}{\mu_{H}},0,0,0,\frac{\Lambda_{V}}{\mu_{V}},0,0\right)$. To obtain the basic reproduction number for the model (1), we follow the method [27], and obtain the following matrices

$$\begin{array}{c} F=\left(\begin{array}{cccc} 0 & \frac{\rho \beta_{H}\Lambda_{H}}{\mu_{H}} & 0 & \frac{\beta_{H}\Lambda_{H}}{\mu_{H}}\\ 0 & 0 & 0 & 0\\ 0 & \frac{\beta_{V}\Lambda_{V}}{\mu_{V}} & 0 & 0\\ 0 & 0 & 0 & 0 \end{array}\right),V=\left(\begin{array}{cccc} k_{1} & 0 & 0 & 0\\ -\chi_{h} & k_{2} & 0 & 0\\ 0 & 0 & k_{3} & 0\\ 0 & 0 & -\delta_{V} & \mu_{V} \end{array}\right). \end{array}$$

where *k*_1_ = *μ*_*H*_ + *χ*_*H*_, *k*_2_ = (*μ*_*H*_ + *γ* + *η*) and *k*_3_ = (*μ*_*V*_ + *δ*_*V*_). The spectral radius of the matrix *ρ*(*FV*^−1^) is the basic reproduction number of the Model (1), given by

$$\begin{array}{c} R_{0}=\frac{\rho \beta_{H}\Lambda_{H}\chi_{H}}{2\mu_{H}k_{1}k_{2}}+\sqrt{\frac{\rho^{2}\beta_{H}^{2}\Lambda_{H}^{2}\chi_{H}^{2}}{4\mu_{H}^{2}k_{1}{}^{2}k_{2}{}^{2}}+\frac{\beta_{H}\Lambda_{H}\chi_{H}\beta_{V}\delta_{V}\Lambda_{V}}{\mu_{H}\mu_{V}^{2}k_{1}k_{2}k_{3}}}=R_{1}+R_{2} \end{array}$$

where

$$\begin{array}{c} R_{1}=\frac{\rho \beta_{H}\Lambda_{H}\chi_{H}}{2\mu_{H}k_{1}k_{2}} \end{array}$$

and

$$\begin{array}{c} R_{2}=\sqrt{\frac{\rho^{2}\beta_{H}^{2}\Lambda_{H}^{2}\chi_{H}^{2}}{4\mu_{H}^{2}k_{1}{}^{2}k_{2}{}^{2}}+\frac{\beta_{H}\Lambda_{H}\chi_{H}\beta_{V}\delta_{V}\Lambda_{V}}{\mu_{H}\mu_{V}^{2}k_{1}k_{2}k_{3}}}. \end{array}$$

### 3.1 Local stability disease free equilibrium

In this subsection we show the local stability of disease free equilibrium *E*_0_. We present the following result:

**Theorem 3.1**. *The disease free equilibrium of model* (1) *is stable locally asymptotically if* $R_{0}<1$, *otherwise unstable*.

**Proof:** The associated Jacobian matrix of the system (1) at $E_{0}=\left(\frac{\Lambda_{H}}{\mu_{H}},0,0,0,\frac{\Lambda_{V}}{\mu_{V}},0,0\right)$ is given by

$$\begin{array}{c} J_{0}=\left(\begin{array}{ccccccc} -\mu_{H} & 0 & -\frac{\rho \beta_{H}\Lambda_{H}}{\mu_{H}} & 0 & 0 & 0 & -\frac{\beta_{H}\Lambda_{H}}{\mu_{H}}\\ 0 & -k_{1} & \frac{\rho \beta_{H}\Lambda_{H}}{\mu_{H}} & 0 & 0 & 0 & \frac{\beta_{H}\Lambda_{H}}{\mu_{H}}\\ 0 & \chi_{H} & -k_{2} & 0 & 0 & 0 & 0\\ 0 & 0 & \gamma & -\mu_{H} & 0 & 0 & 0\\ 0 & 0 & -\frac{\beta_{V}\Lambda_{V}}{\mu_{V}} & 0 & -\mu_{V} & 0 & 0\\ 0 & 0 & \frac{\beta_{V}\Lambda_{V}}{\mu_{V}} & 0 & 0 & -k_{3} & 0\\ 0 & 0 & 0 & 0 & 0 & \delta_{V} & -\mu_{V} \end{array}\right) \end{array}$$

The three eigenvalues of the above jacobian matrix are clearly negative, that is −*μ*_*H*_, −*μ*_*H*_ and −*μ*_*V*_. The remaining four roots can then be determined through the following equation:

$$\begin{array}{c} \lambda^{4}+a_{1}\lambda^{3}+a_{2}\lambda^{2}+a_{3}\lambda +a_{4}=0, \end{array}$$

where

$$\begin{array}{ccc} a_{1} & = & k_{1}+k_{2}+k_{3}+\mu_{V},\\ a_{2} & = & \left(k_{1}+k_{2}+k_{3}\right)\mu_{V}+\left(k_{1}+k_{2}\right)k_{3}+\left(k_{1}k_{2}-\frac{\rho \beta_{H}\Lambda_{H}\chi_{H}}{\mu_{H}}\right),\\ a_{3} & = & \left(k_{3}+\mu_{V}\right)\left(k_{1}k_{2}-\frac{\rho \beta_{H}\Lambda_{H}\chi_{H}}{\mu_{H}}\right)+k_{1}k_{3}\mu_{V}+k_{2}k_{3}\mu_{V},\\ a_{4} & = & k_{1}k_{2}k_{3}\mu_{V}\left(1-R_{0}^{*}\right) \end{array}$$

The above characteristics equation will give four negative eigenvalues if $R_{0}<1$ and the Routh Hurtwiz criteria, *a*_*i*_ > 0, for *i* = 1, 2, 3, 4, and $a_{1}a_{2}a_{3}>a_{1}^{2}a_{4}+a_{3}^{2}$ satisfy. It is obvious that the coefficients *a*_*i*_ for *i* = 1, …4 are clearly positive, if $R_{0}<1$ and $(k_{1}k_{2}-\frac{\rho \beta_{H}\Lambda_{H}\chi_{H}}{\mu_{H}})>0$. Thus, it follows, that the system (1) at the disease free equilibrium *E*_0_ is locally asymptotically stable if $R_{0}<1$.

## 4 Endemic equilibria and bifurcation analysis

The endemic equilibria of the System (1) at $E_{1}=\left(S_{H}^{*},E_{H}^{*},I_{H}^{*},R_{H}^{*},S_{V}^{*},E_{V}^{*},I_{V}^{*}\right)$ is given by

$$\begin{array}{c} \left\{ \begin{array}{c} S_{H}^{*}=\frac{k_{3}\Lambda_{H}\mu_{V}\left(I_{H}^{*}\beta_{V}+\mu_{V}\right)}{k_{3}\mu_{V}\left(\rho \beta_{H}I_{H}^{*}+\mu_{H}\right)\left(I_{H}^{*}\beta_{V}+\mu_{V}\right)+\beta_{H}I_{H}^{*}\beta_{V}\delta_{V}\Lambda_{V}}\\ E_{H}^{*}=\frac{\beta_{H}I_{H}^{*}\Lambda_{H}\left(k_{3}\rho \mu_{V}\left(I_{H}^{*}\beta_{V}+\mu_{V}\right)+\beta_{V}\delta_{V}\Lambda_{V}\right)}{k_{1}\left(k_{3}\mu_{V}\left(\rho \beta_{H}I_{H}^{*}+\mu_{H}\right)\left(I_{H}^{*}\beta_{V}+\mu_{V}\right)+\beta_{H}I_{H}^{*}\beta_{V}\delta_{V}\Lambda_{V}\right)}\\ R_{H}^{*}=\frac{\gamma I_{H}^{*}}{\mu_{H}}\\ S_{V}^{*}=\frac{\Lambda_{V}}{\mu_{V}+I_{H}^{*}\beta_{V}}\\ E_{V}^{*}=\frac{I_{H}^{*}\beta_{V}\Lambda_{V}}{k_{3}\left(\mu_{V}+I_{H}^{*}\beta_{V}\right)}\\ I_{V}^{*}=\frac{I_{H}^{*}\beta_{V}\delta_{V}\Lambda_{V}}{\mu_{V}k_{3}\left(\mu_{V}+I_{H}^{*}\beta_{V}\right)} \end{array}\right. \end{array}$$ (5)

The endemic equilibria Eq (5) satisfies

$$\begin{array}{c} I_{H}^{*}\left(a{I_{H}^{*}}^{2}+bI_{H}^{*}+c\right)=0, \end{array}$$ (6)

where

$$\begin{array}{ccc} a & = & \rho \beta_{H}\beta_{V}\mu_{V}k_{1}k_{2}k_{3},\\ b & = & \beta_{H}\beta_{V}\left(k_{1}k_{2}\delta_{V}\Lambda_{V}-k_{3}\rho \Lambda_{H}\chi_{H}\mu_{V}\right)+k_{1}k_{2}k_{3}\mu_{V}\left(\rho \beta_{H}\mu_{V}+\mu_{H}\beta_{V}\right),\\ c & = & \mu_{H}\mu_{V}^{2}k_{1}k_{2}k_{3}\left(1-R_{0}^{*}\right) \end{array}$$ (7)

where $R_{0}^{*}=R_{0}^{2}+2R_{1}\left(1-R_{0}\right).$ The coefficient *a* in Eq (7) is obviously positive and *c* is positive whenever $R_{0}<1$ and negative if $R_{0}>1$. It is the sign of *b* and *c* will decide about the positive solution of Eq (7). Let $R_{0}>1$, then there exists two roots for Eq (7), one is positive and the other is negative. *c* = 0 if $R_{0}=1$, then a unique non-zero solution exists i.e., $I_{H}^{*}=-b/a$, for *b* < 0. Equilibria depend continually on $R_{0}$ changes which shows that there exists an interval to the left of $R_{0}$ on which there are two positive equilibria

$$\begin{array}{c} {I_{H}^{*}}_{1}=\frac{-b-\sqrt{b^{2}-4ac}}{2a}\;and\;{I_{H}^{*}}_{2}=\frac{-b+\sqrt{b^{2}-4ac}}{2a}. \end{array}$$

There is no positive solution of Eq (7) if *c* > 0 and either *b* ≥ 0 or *b*^2^ < 4*ac* and hence no endemic equilibria. We establish the following:

**Theorem 4.1**. *The system* (1) *has*:

1. *if* *c* < 0 *if and only if* $R_{0}>1$ *then a unique endemic equilibrium exists*;
2. *if* *b* < 0 *and* *c* = 0 *or* *b*^2^ − 4*ac* = 0 *then a unique endemic equilibrium exists*;
3. *if* *c* > 0 *and* *b* < 0 *and* *b*^2^ − 4*ac* > 0 *then two equilibria exists*;
4. *otherwise no endemic equilibrium*.

Case (iii) of (4.1) shows the possibility of a backward bifurcation in system (1) when $R_{0}<1$. The backward bifurcation can be obtained by setting *b*^2^ − 4*ac* = 0 and solved for the critical value of $R_{0}$, shown by $R_{c}$ is given by

$$\begin{array}{c} R_{c}=1-\frac{b^{2}}{4a\mu_{H}\mu_{V}^{2}k_{1}k_{2}k_{3}}. \end{array}$$

**Fig 1:**
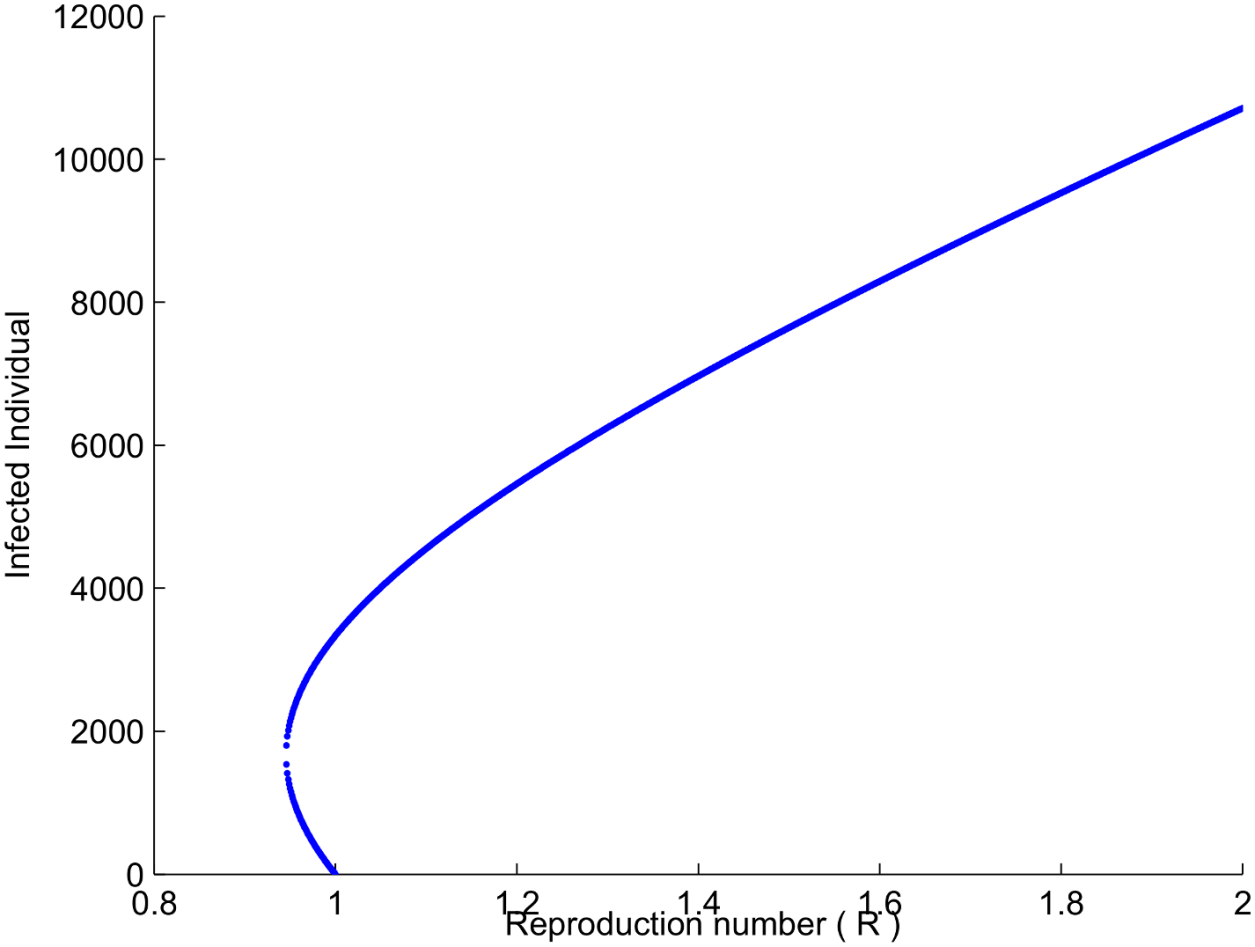
The plot shows the bifurcation diagram.

Thus, $R_{c}<R_{0}$ is equivalent to *b*^2^ − 4*ac* > 0 and therefore, backward bifurcation would occur for values of $R_{0}$ such that $R_{c}<R_{0}<1$. This fact can be seen for choosing the parameters values of the model (1):*χ*_*H*_ = 0.0022, *β*_*H*_ = 0.0002, *β*_*V*_ = 0.0009, *μ*_*H*_ = 0.01, *δ*_*V*_ = 0.3, *μ*_*V*_ = 0.003, Λ_*V*_ = 1.3, Λ_*H*_ = 0.4, *η* = 0.11, *ρ* = 0.029, *γ* = 0.0614799. The bifurcation diagram is presented in Fig 1, which demonstrate the existence of two locally asymptotically stable equilibria whenever $R_{0}<1$, which confirm the occupance of a backward bifurcation in system (1).

### 4.1 Existence of bifurcation

In order to establish the backward bifurcation phenomenon, we use the centre manifold theory [9, 28]. We take into account the transmission rate, *β*_*H*_ as bifurcation parameter so that $R_{0}^{*}=1$ if and only if $\beta_{H}^{*}=\beta_{H}=\frac{k_{1}k_{2}k_{3}\mu_{H}\mu_{V}^{2}}{\Lambda_{H}\chi_{H}\left(k_{3}\rho \mu_{V}^{2}+\beta_{V}\delta_{V}\Lambda_{V}\right)}$. The following variations are made in the variables of the system (1) so that *S*_*H*_ = *x*_1_, *E*_*H*_ = *x*_2_, *I*_*H*_ = *x*_3_, *R*_*H*_ = *x*_4_, *S*_*V*_ = *x*_5_, *E*_*V*_ = *x*_6_, *I*_*V*_ = *x*_7_. Also, further adopting vector notation *x* = (*x*_1_, *x*_2_, *x*_3_, *x*_4_, *x*_5_, *x*_6_, *x*_7_)^*T*^. Zika model can then be formulated in the form $\frac{dx}{dt}=F\left(x\right)$, with *F* = (*f*_1_, *f*_2_, *f*_3_, *f*_4_, *f*_5_, *f*_6_, *f*_7_)^*T*^ as indicated below:

$$\begin{array}{c} \begin{array}{c} \frac{dx_{1}}{dt}=\Lambda_{H}-\beta_{H}x_{1}\left(x_{7}+\rho x_{3}\right)-\mu_{H}x_{1}\\ \frac{dx_{2}}{dt}=\beta_{H}x_{1}\left(x_{7}+\rho x_{3}\right)-k_{1}x_{2}\\ \frac{dx_{3}}{dt}=\chi_{H}x_{2}-k_{2}x_{3}\\ \frac{dx_{4}}{dt}=\gamma x_{3}-\mu_{H}x_{4}\\ \frac{dx_{5}}{dt}=\Lambda_{V}-\beta_{V}x_{3}x_{5}-\mu_{V}x_{5}\\ \frac{dx_{6}}{dt}=\beta_{V}x_{3}x_{5}-k_{3}x_{6}\\ \frac{dx_{7}}{dt}=\delta_{V}x_{6}-\mu_{V}x_{7} \end{array}. \end{array}$$ (8)

The system (8) is evaluated at the Jacobian matrix for the disease free endemic state (DFE) *E*_0_ denoted by *J*(*E*_0_) gives

$$\begin{array}{c} J\left(E_{0}\right)=\left(\begin{array}{ccccccc} -\mu_{H} & 0 & -\frac{\rho \beta_{H}\Lambda_{H}}{\mu_{H}} & 0 & 0 & 0 & -\frac{\beta_{H}\Lambda_{H}}{\mu_{H}}\\ 0 & -k_{1} & \frac{\rho \beta_{H}\Lambda_{H}}{\mu_{H}} & 0 & 0 & 0 & \frac{\beta_{H}\Lambda_{H}}{\mu_{H}}\\ 0 & \chi_{H} & -k_{2} & 0 & 0 & 0 & 0\\ 0 & 0 & \gamma & -\mu_{H} & 0 & 0 & 0\\ 0 & 0 & \frac{\mu_{V}k_{3}\left(\rho \beta_{H}\Lambda_{H}\chi_{H}-\mu_{H}k_{2}k_{1}\right)}{\beta_{H}\delta_{V}\Lambda_{H}\chi_{H}} & 0 & -\mu_{V} & 0 & 0\\ 0 & 0 & \frac{\mu_{V}k_{3}\left(\mu_{H}k_{2}k_{1}-\rho \beta_{H}\Lambda_{H}\chi_{H}\right)}{\beta_{H}\delta_{V}\Lambda_{H}\chi_{H}} & 0 & 0 & -k_{3} & 0\\ 0 & 0 & 0 & 0 & 0 & \delta_{V} & -\mu_{V} \end{array}\right) \end{array}$$

has a simple zero eigenvalue, at the other eigenvalues having negative real parts. Hence, the Center Manifold theorem [28] can be applied. For this we need to calculate *a* and *b*. We first start by calculating the right and the left eigenvector of *J*(*E*_0_) denoted respectively by **W** = [*w*_1_, *w*_2_, *w*_3_, *w*_4_, *w*_5_, *w*_6_, *w*_7_]^*T*^ and **V** = [*v*_1_, *v*_2_, *v*_3_, *v*_4_, *v*_5_, *v*_6_, *v*_7_]. We obtain

$$\begin{array}{ccc} w_{1} & = & -\frac{w_{2}k_{1}}{\mu_{H}},\;w_{2}>0,\;w_{3}=\frac{w_{2}\chi_{H}}{kk_{2}},\;w_{4}=\frac{\gamma w_{2}\chi_{H}}{\mu_{H}k_{2}},\;w_{5}=\frac{w_{2}k_{3}\left(\rho \beta_{H}\Lambda_{H}\chi_{H}-\mu_{H}k_{1}k_{2}\right)}{\beta_{H}\Lambda_{H}\delta_{V}k_{2}},\\ w_{6} & = & -\frac{w_{2}\mu_{V}\left(\frac{\rho \chi_{H}}{k_{2}}-\frac{\mu_{H}k_{1}}{\beta_{H}\Lambda_{H}}\right)}{\delta_{V}},\;w_{7}=-w_{2}\left(\frac{\rho \chi_{H}}{k_{2}}-\frac{\mu_{H}k_{1}}{\beta_{H}\Lambda_{H}}\right) \end{array}$$

and

$$\begin{array}{ccc} v_{1} & = & v_{4}=v_{5}=0,\;v_{6}>0,\;v_{2}=\frac{v_{6}\mu_{H}\left(\delta_{V}\mu_{V}+\mu_{V}^{2}\right)}{\beta_{H}\Lambda_{H}\delta_{V}},\;\\ v_{3} & = & \frac{v_{6}\mu_{H}kk_{1}\left(kk_{3}+\mu_{V}^{2}\right)}{\beta_{H}\Lambda_{H}\chi_{H}\delta_{V}},\;v_{7}=\frac{v_{6}k_{3}}{\delta_{V}}. \end{array}$$

After some rigorous algebra computations, it can be shown that

$$\begin{array}{c} a=-\frac{2v_{2}w_{2}^{2}\left(-\frac{\beta_{H}^{3}k_{1}\left(\frac{\rho \chi_{H}}{k_{2}}-\frac{\mu_{H}k_{1}}{\beta_{H}\Lambda_{H}}\right)}{\mu_{H}}+\frac{\rho \beta_{H}^{3}\chi_{H}k_{1}}{\mu_{H}k_{2}}+\frac{v_{6}\mu_{V}^{2}k_{3}{}^{2}\left(\rho \beta_{H}\Lambda_{H}\chi_{H}-\mu_{H}k_{1}k_{3}\right){}^{2}}{\Lambda_{H}^{2}\delta_{V}^{2}\Lambda_{V}k_{2}{}^{2}}\right)}{\beta_{H}^{2}} \end{array}$$

and

$$\begin{array}{c} b=\frac{v_{2}w_{2}\Lambda_{H}\left(\frac{\rho \mu_{H}k_{1}}{\beta_{H}\Lambda_{H}}-\frac{\left(\rho^{2}-1\right)\chi_{H}}{k_{2}}\right)}{\mu_{H}} \end{array}$$

Here, the coefficient *b* is positive, it is the sign of *b* will determine the occurrence of backward bifurcation in the given model. It follows from Theorem [28] that the system (1) will undergo backward bifurcation if the coefficient *a* is positive. This is implying that the disease free is not globally stable.

### 4.2 Local stability endemic equilibrium

**Theorem 4.2**. *The endemic equilibrium of the model* (1) *is locally asymptotically stable, if* $R_{0}>1$.

**Proof:** *At* *E*_1_ *without* *R*_*H*_ *the jacobian matrix evaluated as*

$$\begin{array}{c} J\left(E_{1}\right)=\left(\begin{array}{cccccc} -k_{4}-\mu_{H} & 0 & -k_{5} & 0 & 0 & -k_{8}\\ k_{4} & -k_{1} & k_{5} & 0 & 0 & k_{8}\\ 0 & \chi_{H} & -k_{2} & 0 & 0 & 0\\ 0 & 0 & -k_{6} & -k_{7}-\mu_{V} & 0 & 0\\ 0 & 0 & k_{6} & k_{7} & -k_{3} & 0\\ 0 & 0 & 0 & 0 & \delta_{V} & -\mu_{V} \end{array}\right), \end{array}$$

*where* $k_{4}=\beta_{H}\left(\rho I_{H}^{*}+I_{V}^{*}\right),\;\;k_{5}=\rho \beta_{H}S_{H}^{*},\;\;k_{6}=S_{V}^{*}\beta_{V},\;\;k_{7}=I_{H}^{*}\beta_{V},\;\;k_{8}=\beta_{H}S_{H}^{*}.$

*The associate characteristics equation of* *J*(*E*_1_) *is*

$$\begin{array}{c} \lambda^{6}+c_{1}\lambda^{5}+c_{2}\lambda^{4}+c_{3}\lambda^{3}+c_{4}\lambda^{2}+c_{5}\lambda +c_{6}=0, \end{array}$$ (9)

*where*

$$\begin{array}{ccc} c_{1} & = & \mu_{H}+k_{1}+k_{2}+k_{3}+k_{4}+k_{7}+2\mu_{V},\\ c_{2} & = & k_{3}k_{4}+\left(k_{3}+k_{4}\right)k_{7}+k_{2}\left(k_{3}+k_{4}+k_{7}\right)+k_{1}\left(k_{2}+k_{3}+k_{4}+k_{7}\right)+\\ & & \left(k_{1}+k_{2}+k_{3}+k_{7}\right)\mu_{H}+\mu_{V}\left(2\mu_{H}+2k_{1}+2k_{2}+2k_{3}+2k_{4}+k_{7}\right)-k_{5}\chi_{H}+\mu_{V}^{2},\\ c_{3} & = & k_{3}k_{7}\mu_{H}-k_{5}\chi_{H}k_{9}+2k_{3}\mu_{H}\mu_{V}+k_{7}\mu_{H}\mu_{V}+k_{2}\left(k_{7}\left(\mu_{H}+k_{4}\right)+\mu_{V}k_{10}+k_{3}k_{11}\right)\\ & & +k_{2}\mu_{V}^{2}+k_{1}\left(k_{7}\left(\mu_{H}+k_{4}\right)+\mu_{V}k_{10}+k_{3}k_{11}+k_{2}\left(k_{3}+k_{11}\right)+\mu_{V}^{2}\right)\\ & & +\mu_{V}^{2}k_{12}+k_{3}\mu_{V}\left(2k_{4}+k_{7}\right)+k_{4}k_{7}\left(\mu_{V}+k_{3}\right),\\ c_{4} & = & -\chi_{H}\left(k_{5}\left(k_{7}\left(\mu_{H}+\mu_{V}\right)+k_{3}\left(\mu_{H}+k_{7}+2\mu_{V}\right)+\mu_{V}\left(2\mu_{H}+\mu_{V}\right)\right)+k_{6}k_{8}\delta_{V}\right)\\ & & +k_{1}\left(k_{2}\left(\mu_{V}\left(2\mu_{H}+2k_{4}+k_{7}\right)+k_{3}k_{11}+k_{7}k_{13}+\mu_{V}^{2}\right)+k_{13}k_{14}\mu_{V}\right)+k_{3}k_{13}k_{14}\mu_{V}\\ & & +k_{1}k_{3}\left(k_{10}\mu_{V}+k_{7}k_{13}+\mu_{V}^{2}\right)+k_{2}\left(k_{13}k_{14}\mu_{V}+k_{3}\left(k_{10}\mu_{V}+k_{7}k_{13}+\mu_{V}^{2}\right)\right),\\ c_{5} & = & -\chi_{H}\left[k_{6}k_{8}\delta_{V}\left(\mu_{H}+\mu_{V}\right)+k_{5}k_{14}\mu_{H}\mu_{V}+k_{3}k_{5}\left(k_{7}\left(\mu_{H}+\mu_{V}\right)+\mu_{V}\left(2\mu_{H}+\mu_{V}\right)\right)\right]\\ & & +k_{1}\left(k_{2}\left(k_{3}\left(\mu_{V}\left(2\mu_{H}+2k_{4}+k_{7}\right)+k_{7}k_{13}+\mu_{V}^{2}\right)+k_{13}k_{14}\mu_{V}\right)+k_{3}k_{13}k_{14}\mu_{V}\right)\\ & & +k_{2}k_{3}k_{13}k_{14}\mu_{V},\\ c_{6} & = & \mu_{V}\left(k_{1}k_{2}k_{3}k_{13}k_{14}-\mu_{H}\chi_{H}\left(k_{6}k_{8}\delta_{V}+k_{3}k_{5}\left(k_{7}+\mu_{V}\right)\right)\right), \end{array}$$

*where* *k*_9_ = (*μ*_*H*_ + *k*_3_ + *k*_7_ + 2*μ*_*V*_), *k*_10_ = (2*μ*_*H*_ + 2*k*_4_ + *k*_7_), *k*_11_ = (*μ*_*H*_ + *k*_4_ + *k*_7_ + 2*μ*_*V*_), *k*_12_ = (*μ*_*H*_ + *k*_3_ + *k*_4_), *k*_13_ = (*μ*_*H*_ + *k*_4_), *k*_14_ = (*k*_7_ + *μ*_*V*_).

*The Routh-Hurtwiz criteria for Polynomial* Eq (9) *will give six negative eigenvalues if the conditions given below are satisfied*: *C*_*i*_ > 0, *for* *i* = 1, 2, 3, …, 6. *The relevant Routh Hurtwiz criteria in* [29] *could be used to show that the model* (1) *is stable locally asymptotically when* $R_{0}>1$

## 5 Global stability

This section investigates the global results for the model (1) at *E*_0_ and *E*_1_. First, we give the proof of the disease free global stability.

### 5.1 Global stability disease free equilibrium

**Theorem 5.1**. *For* $R_{0}<1$, *the disease free equilibrium* *E*_0_ *of the system* (1) *is globally asymptotically stable*.

**Proof:** To show this result, we define the following lyapunov function

$$\begin{array}{ccc} L(t) & = & w_{1}(S_{H}-S_{H}^{0}-S_{H}^{0}log\frac{S_{H}}{S_{H}^{0}})+w_{2}E_{H}+w_{3}I_{H}+w_{4}(S_{V}-S_{V}^{0}-S_{V}^{0}log\frac{S_{V}}{S_{V}^{0}})\\ & & +w_{5}E_{V}+w_{6}I_{V}. \end{array}$$

Taking the time derivative of *L*(*t*), we have

$$\begin{array}{c} L\left(t\right)=w_{1}(1-\frac{S_{H}^{0}}{S_{H}})S_{H}^{\prime }+w_{2}E_{H}^{\prime }+w_{3}I_{H}^{\prime }+w_{4}(1-\frac{S_{V}^{0}}{S_{V}})S_{V}^{\prime }+w_{5}E_{V}^{\prime }+w_{6}I_{V}^{\prime }. \end{array}$$

Using system (1), we have

$$\begin{array}{ccc} L(t) & = & w_{1}(1-\frac{S_{H}^{0}}{S_{H}})\left[\Lambda_{H}-\beta_{H}S_{H}\left(I_{V}+\rho I_{H}\right)-\mu_{H}S_{H}\right]\\ & & +w_{2}\left[\beta_{H}S_{H}\left(I_{V}+\rho I_{H}\right)-\left(\mu_{H}+\chi_{H}\right)E_{H}\right]\\ & & +w_{3}\left[\chi_{H}E_{H}-\left(\mu_{H}+\gamma +\eta \right)I_{H}\right]+w_{4}(1-\frac{S_{V}^{0}}{S_{V}})\left[\Lambda_{V}-\beta_{V}S_{V}I_{H}-\mu_{V}S_{V}\right]\\ & & +w_{5}\left[\beta_{V}S_{V}I_{H}-\left(\mu_{V}+\delta_{V}\right)E_{V}\right]+w_{6}\left[\delta_{V}E_{V}-\mu_{V}I_{V}\right]. \end{array}$$

Using $S_{H}^{0}=\frac{\Lambda_{H}}{\mu_{H}}$, $S_{V}^{0}=\frac{\Lambda_{V}}{\mu_{V}}$, and after simplifications, we obtain

$$\begin{array}{ccc} L(t) & = & -\mu_{H}w_{1}\frac{{\left(S_{H}-S_{H}^{0}\right)}^{2}}{S_{H}}+\left[w_{2}-w_{1}\right]\beta_{H}S_{H}\left(I_{V}+\rho I_{H}\right)\\ & & +\left[w_{3}\chi_{H}-w_{2}\left(\chi_{H}+\mu_{H}\right)\right]E_{H}+\left[w_{1}\beta_{H}\frac{\Lambda_{H}}{\mu_{H}}\rho +w_{4}\beta_{V}\frac{\Lambda_{V}}{\mu_{V}}-w_{3}\left(\mu_{H}+\gamma +\eta \right)\right]I_{H}\\ & & +\left[w_{5}-w_{4}\right]\beta_{V}S_{V}I_{H}-\mu_{V}w_{4}\frac{{\left(S_{V}-S_{V}^{0}\right)}^{2}}{S_{V}}\\ & & +\left[w_{6}\delta_{V}-w_{5}\left(\mu_{V}+\delta_{V}\right)\right]E_{V}+\left[w_{1}\beta_{H}\frac{\Lambda_{H}}{\mu_{H}}-w_{6}\mu_{v}\right]I_{V}. \end{array}$$

Let’s choose the constants: *w*_1_ = *w*_2_ = *χ*_*H*_, $w_{4}=w_{5}=\frac{\delta_{V}\beta_{H}\chi_{H}\Lambda_{H}}{\mu_{H}\mu_{V}\left(\delta_{V}+\mu_{V}\right)}$, *w*_3_ = (*μ*_*H*_+*χ*_*H*_), $w_{6}=\frac{\beta_{H}\chi_{H}\Lambda_{H}}{\mu_{V}\mu_{H}}$, we get

$$\begin{array}{ccc} L^{\prime }\left(t\right) & = & -\chi_{H}\mu_{H}\frac{{\left(S_{H}-S_{H}^{0}\right)}^{2}}{S_{H}}-\frac{\delta_{V}\beta_{H}\chi_{H}\Lambda_{H}}{\mu_{H}\left(\delta_{V}+\mu_{V}\right)}\frac{{\left(S_{V}-S_{V}^{0}\right)}^{2}}{S_{V}}\\ & & -\left(\mu_{H}+\chi_{H}\right)\left(\mu_{H}+\gamma +\eta \right)(1-R_{0}^{*})I_{H}. \end{array}$$

Thus, *L*′(*t*) is negative for $R_{0}\leq 1$ and zero if and only if $S_{H}=S_{H}^{0}$, $S_{V}=S_{V}^{0}$, *E*_*H*_ = *I*_*H*_ = *R*_*H*_ = 0 and *E*_*V*_ = *I*_*V*_ = 0. Therefore the largest compact invariant set in *Π* is the singleton set *E*_0_. So, the model (1) is globally asymptotically stable.

### 5.2 Global stability endemic equilibrium

Before, we proceed to obtain the global stability of the model (1) at endemic equilibrium *E*_1_, first, at endemic steady state we, obtain:

$$\begin{array}{c} \left\{ \begin{array}{c} \Lambda_{H}=\beta_{H}S_{H}^{*}\left(I_{V}^{*}+\rho I_{H}^{*}\right)+\mu_{H}S_{H}^{*}\\ \left(\mu_{H}+\chi_{H}\right)E_{H}^{*}=\beta_{H}S_{H}^{*}\left(I_{V}^{*}+\rho I_{H}^{*}\right)\\ \chi_{H}E_{H}^{*}=\left(\mu_{H}+\gamma +\eta \right)I_{H}^{*}\\ \frac{\left(\chi_{H}+\mu_{H}\right)\left(\mu_{H}+\gamma +\eta \right)}{\chi_{H}}I_{H}^{*}=\beta_{H}S_{H}^{*}\left(I_{V}^{*}+\rho I_{H}^{*}\right)\\ \Lambda_{V}=\beta_{V}S_{V}^{*}I_{H}^{*}+\mu_{V}S_{V}^{*}\\ \beta_{V}S_{V}^{*}I_{H}^{*}=\left(\mu_{V}+\delta_{V}\right)E_{V}^{*}\\ \delta_{V}E_{V}^{*}=\mu_{V}I_{V}^{*}\\ \beta_{V}S_{V}^{*}I_{H}^{*}=\frac{\left(\mu_{V}+\delta_{V}\right)\mu_{V}I_{V}^{*}}{\delta_{V}} \end{array}\right. \end{array}$$

Now, we prove the global stability of the model (1) at endemic equilibrium *E*_1_ by following [30–32].

**Theorem 5.2**. *If* $R_{0}>1$, *then the endemic equilibrium* *E*_1_ *is globally asymptotically stable*.

**Proof:** Consider the lyapunove function:

$$\begin{array}{ccc} L & = & \int_{S_{H}^{*}}^{S_{H}}\left(1-\frac{S_{H}^{*}}{x}\right)dx+\int_{E_{H}^{*}}^{E_{H}}\left(1-\frac{E_{H}^{*}}{x}\right)dx+\frac{\left(\mu_{H}+\chi_{H}\right)}{\chi_{H}}\int_{I_{H}^{*}}^{I_{H}}\left(1-\frac{I_{H}^{*}}{x}\right)dx\\ & & +\int_{S_{V}^{*}}^{S_{V}}\left(1-\frac{S_{V}^{*}}{x}\right)dx+\int_{E_{V}^{*}}^{E_{V}}\left(1-\frac{E_{V}^{*}}{x}\right)dx+\frac{\left(\mu_{V}+\delta_{V}\right)}{\delta_{V}}\int_{I_{V}^{*}}^{I_{V}}\left(1-\frac{I_{V}^{*}}{x}\right)dx. \end{array}$$

The derivative of *L* along the solutions of system (1) is

$$\begin{array}{ccc} \dot{L} & = & (1-\frac{S_{H}^{*}}{S_{H}})S_{H}^{\prime }+(1-\frac{E_{H}^{*}}{E_{H}})E_{H}^{\prime }+\frac{\left(\mu_{H}+\chi_{H}\right)}{\chi_{H}}(1-\frac{I_{H}^{*}}{I_{H}})I_{H}^{\prime }+(1-\frac{S_{V}^{*}}{S_{V}})S_{V}^{\prime }\\ & & +(1-\frac{E_{V}^{*}}{E_{V}})E_{V}^{\prime }+\frac{\left(\mu_{V}+\delta_{V}\right)}{\delta_{V}}(1-\frac{I_{V}^{*}}{I_{V}})I_{V}^{\prime }. \end{array}$$

By direct calculations, we have that:

$$\begin{array}{ccc} \left(1-\frac{S_{H}^{*}}{S_{H}}\right)S_{H}^{\prime } & = & \left(1-\frac{S_{H}^{*}}{S_{H}}\right)\left[\Lambda_{H}-\beta_{H}S_{H}\left(I_{V}+\rho I_{H}\right)-\mu_{H}S_{H}\right]\\ & = & \left(1-\frac{S_{H}^{*}}{S_{H}}\right)\left[\beta_{H}S_{H}^{*}\left(I_{V}^{*}+\rho I_{H}^{*}\right)-\beta_{H}S_{H}\left(I_{V}+\rho I_{H}\right)+\mu_{H}S_{H}^{*}-\mu_{H}S_{H}\right]\\ & = & \mu_{H}S_{H}^{*}\left(2-\frac{S_{H}}{S_{H}^{*}}-\frac{S_{H}^{*}}{S_{H}}\right)+\left(1-\frac{S_{H}^{*}}{S_{H}}\right)\beta_{H}S_{H}^{*}\left(I_{V}^{*}+\rho I_{H}^{*}\right)\\ & & -\beta_{H}S_{H}\left(I_{V}+\rho I_{H}\right)+\beta_{H}S_{H}^{*}\left(I_{V}+\rho I_{H}\right), \end{array}$$ (10)

$$\begin{array}{ccc} \left(1-\frac{E_{H}^{*}}{E_{H}}\right)E_{H}^{\prime } & = & \left(1-\frac{E_{H}^{*}}{E_{H}}\right)\left[\beta_{H}S_{H}\left(I_{V}+\rho I_{H}\right)-\left(\mu_{H}+\chi_{H}\right)E_{H}\right]\\ & = & \beta_{H}S_{H}\left(I_{V}+\rho I_{H}\right)-\beta_{H}S_{H}\left(I_{V}+\rho I_{H}\right)\frac{E_{H}^{*}}{E_{H}}\\ & & -\left(\mu_{H}+\chi_{H}\right)E_{H}+\left(\mu_{H}+\chi_{H}\right)E_{H}^{*}\\ & = & \beta_{H}S_{H}\left(I_{V}+\rho I_{H}\right)-\beta_{H}S_{H}\left(I_{V}+\rho I_{H}\right)\frac{E_{H}^{*}}{E_{H}}-\left(\mu_{H}+\chi_{H}\right)E_{H}\\ & & +\beta_{H}S_{H}^{*}\left(I_{V}^{*}+\rho I_{H}^{*}\right), \end{array}$$ (11)

$$\begin{array}{ccc} \left(1-\frac{I_{H}^{*}}{I_{H}}\right)\frac{\left(\mu_{H}+\chi_{H}\right)}{\chi_{H}}I_{H}^{\prime } & = & \left(1-\frac{I_{H}^{*}}{I_{H}}\right)\frac{\left(\mu_{H}+\chi_{H}\right)}{\chi_{H}}\left[\chi_{H}E_{H}-\left(\mu_{H}+\gamma +\eta \right)I_{H}\right]\\ & = & \left(\mu_{H}+\chi_{H}\right)E_{H}-\left(\mu_{H}+\chi_{H}\right)E_{H}\frac{I_{H}^{*}}{I_{H}}\\ & & -\frac{\left(\mu_{H}+\chi_{H}\right)\left(\mu_{H}+\gamma +\eta \right)}{\chi_{H}}I_{H}\\ & & +\frac{\left(\mu_{H}+\chi_{H}\right)\left(\mu_{H}+\gamma +\eta \right)}{\chi_{H}}I_{H}^{*}\\ & = & \left(\mu_{H}+\chi_{H}\right)E_{H}-\beta_{H}S_{H}^{*}\left(I_{V}^{*}+\rho I_{H}^{*}\right)\frac{E_{H}}{E_{H}^{*}}\frac{I_{H}^{*}}{I_{H}}-\\ & & \beta_{H}S_{H}^{*}\left(I_{V}^{*}+\rho I_{H}^{*}\right)\frac{I_{H}}{I_{H}^{*}}\\ & & +\beta_{H}S_{H}^{*}\left(I_{V}^{*}+\rho I_{H}^{*}\right), \end{array}$$ (12)

$$\begin{array}{ccc} \left(1-\frac{S_{V}^{*}}{S_{V}}\right)S_{V}^{\prime } & = & \left(1-\frac{S_{V}^{*}}{S_{V}}\right)\left[\Lambda_{V}-\beta_{V}S_{V}I_{H}-\mu_{V}S_{V}\right]\\ & = & \left(1-\frac{S_{V}^{*}}{S_{V}}\right)\left[\beta_{V}S_{V}^{*}I_{H}^{*}+\mu_{V}S_{V}^{*}-\beta_{V}S_{V}I_{H}-\mu_{V}S_{V}\right]\\ & = & \mu_{V}S_{V}^{*}\left(2-\frac{S_{V}}{S_{V}^{*}}-\frac{S_{V}^{*}}{S_{V}}\right)+\left(1-\frac{S_{V}^{*}}{S_{V}}\right)\beta_{V}S_{V}^{*}I_{H}^{*}-\beta_{V}S_{V}I_{H}\\ & & +\beta_{V}S_{V}^{*}I_{H}, \end{array}$$ (13)

$$\begin{array}{ccc} \left(1-\frac{E_{V}^{*}}{E_{V}}\right)E_{V}^{\prime } & = & \left(1-\frac{E_{V}^{*}}{E_{V}}\right)\left[\beta_{V}S_{V}I_{H}-\left(\mu_{V}+\delta_{V}\right)E_{V}\right]\\ & = & \beta_{V}S_{V}I_{H}-\beta_{V}S_{V}I_{H}\frac{E_{V}^{*}}{E_{V}}-\left(\mu_{V}+\delta_{V}\right)E_{V}+\left(\mu_{V}+\delta_{V}\right)E_{V}^{*}\\ & = & \beta_{V}S_{V}I_{H}-\beta_{V}S_{V}I_{H}\frac{E_{V}^{*}}{E_{V}}-\left(\mu_{V}+\delta_{V}\right)E_{V}+\beta_{V}S_{V}^{*}I_{H}^{*}, \end{array}$$ (14)

$$\begin{array}{ccc} \left(1-\frac{I_{V}^{*}}{I_{V}}\right)\frac{\left(\mu_{V}+\delta_{V}\right)}{\delta_{V}}I_{V}^{\prime } & = & \left(1-\frac{I_{V}^{*}}{I_{V}}\right)\frac{\left(\mu_{V}+\delta_{V}\right)}{\delta_{V}}\left[\delta_{V}E_{V}-\mu_{V}I_{V}\right]\\ & = & \left(\mu_{V}+\delta_{V}\right)E_{V}-\left(\mu_{V}+\delta_{V}\right)E_{V}\frac{I_{V}^{*}}{I_{V}}-\frac{\left(\mu_{V}+\delta_{V}\right)\mu_{V}}{\delta_{V}}I_{V}\\ & & +\frac{\left(\mu_{V}+\delta_{V}\right)\mu_{V}}{\delta_{V}}I_{V}^{*}\\ & = & \left(\mu_{V}+\delta_{V}\right)E_{V}-\frac{\beta_{V}S_{V}^{*}I_{H}^{*}}{E_{V}^{*}}E_{V}\frac{I_{V}^{*}}{I_{V}}-\frac{\beta_{V}S_{V}^{*}I_{H}^{*}}{I_{V}^{*}}I_{V}\\ & & +\beta_{V}S_{V}^{*}I_{H}^{*}. \end{array}$$ (15)

It follows from Eqs (10)–(15)

$$\begin{array}{ccc} L & = & \mu_{H}S_{H}^{*}\left(2-\frac{S_{H}}{S_{H}^{*}}-\frac{S_{H}^{*}}{S_{H}}\right)+\mu_{V}S_{V}^{*}\left(2-\frac{S_{V}}{S_{V}^{*}}-\frac{S_{V}^{*}}{S_{V}}\right)\\ & & +\beta_{H}S_{H}^{*}\left(I_{V}^{*}+\rho I_{H}^{*}\right)(3-\frac{S_{H}^{*}}{S_{H}}-\frac{I_{H}}{I_{H}^{*}}-\frac{I_{H}^{*}E_{H}}{E_{H}^{*}I_{H}}+\frac{\left(I_{V}+\rho I_{H}\right)}{\left(I_{V}^{*}+\rho I_{H}^{*}\right)}(1-\frac{S_{H}E_{H}^{*}}{S_{H}^{*}E_{H}}))\\ & & +\beta_{V}S_{V}^{*}I_{H}^{*}(3-\frac{S_{V}^{*}}{S_{V}}-\frac{I_{V}}{I_{V}^{*}}-\frac{E_{V}I_{V}^{*}}{E_{V}^{*}I_{V}}+\frac{I_{H}}{I_{H}^{*}}(1-\frac{S_{V}E_{V}^{*}}{E_{V}S_{V}^{*}}))\;\;\; \end{array}$$ (16)

In Eq (16),

$$\begin{array}{ccc} \left(2-\frac{S_{H}}{S_{H}^{*}}-\frac{S_{H}^{*}}{S_{H}}\right) & \leq & 0,\\ \;\;(2-\frac{S_{V}}{S_{V}^{*}}-\frac{S_{V}^{*}}{S_{V}}) & \leq & 0,\\ \left(3-\frac{S_{H}^{*}}{S_{H}}-\frac{I_{H}}{I_{H}^{*}}-\frac{I_{H}^{*}E_{H}}{E_{H}^{*}I_{H}}+\frac{\left(I_{V}+\rho I_{H}\right)}{\left(I_{V}^{*}+\rho I_{H}^{*}\right)}(1-\frac{S_{H}E_{H}^{*}}{S_{H}^{*}E_{H}})\right) & \leq & 0,\\ \left(3-\frac{S_{V}^{*}}{S_{V}}-\frac{I_{V}}{I_{V}^{*}}-\frac{E_{V}I_{V}^{*}}{E_{V}^{*}I_{V}}+\frac{I_{H}}{I_{H}^{*}}(1-\frac{S_{V}E_{V}^{*}}{E_{V}S_{V}^{*}})\right) & \leq & 0. \end{array}$$

One can see that the largest invariant subset, $\dot{L}=0$ is *E*_1_. So, by LaSalle’s invariance Principle [33], *E*_1_ is globally asymptotically stable whenever $R_{0}>1$.

## 6 Sensitivity analysis of $R_{0}$

We performed sensitivity analysis to explore the model robustness to parameter values used. This is to provide information on the parameters that have significant impact of theoretical model for Zika virus transmission in relation to the reproduction number $R_{0}$. In order to undertake this activity we made use of normalised forward sensitivity index of a variable to a parameter approach vividly described in [34, 35] this technique is expressed as the ratio of the relative variation in the variable to the relative variation in the parameter. It can also be viewed as a differentiable function of the parameter.

**Definition 6.1** *The normalized forward sensitivity index of a variable* *h*, *that depends differentially on a parameter l, is defined as*:

$$\begin{array}{c} {ϒ}_{l}^{h}\equiv \frac{\partial h}{\partial l}\times \frac{l}{h}=1 \end{array}$$ (17)

The detailed sensitivity indices of $R_{0}$ based on the evaluation to the other parameters of the model are presented in Table 1. The parameters are organized in such way that it begins from the most sensitive to the least sensitive one. The most sensitive ones from the Table 1 are the human infected treatment rate, natural death in humans, recruitment rate of humans, the rate of exposed humans moving into infectious class, probability of mosquitoes getting infected, mosquitoes recruitment rate, the rate flow from *E*_*v*_ to *I*_*v*_, human recovery rate due to treatment (*η*, *μ*_*H*_, Λ_*H*_, *χ*_*H*_, *β*_*H*_, *β*_*V*_, Λ_*V*_, *δ*_*V*_ and *γ*, respectively) and the least parameter is the human modification parameter *ρ*.

**Table 1:** Sensitivity indices of $R_{0}$ expressed in terms of $R_{0}$.

	Parameter	Description	Sensitivity
1	*η*	Human infected treatment rate	−0.9004
2	*μ*_*H*_	Natural death rate in human	−0.5710
3	Λ_*H*_	Recruitment rate of humans	−0.5509
4	*χ*_*H*_	The rate of exposed humans moving into infectious class	0.5508
5	*β*_*H*_	Probability of humans getting infected	0.4630
6	*β*_*V*_	Probability of mosquitoes getting infected	0.4490
7	Λ_*V*_	Mosquito recruitment rate	0.4490
8	*δ*_*V*_	The rate flow from *E*_*V*_ to *I*_*V*_	0.2641
9	*γ*	Human recovery rate due to treatment	−0.1809
10	*ρ*	Human modification parameter	0.0146

For instance, increasing (or decreasing) the human infected treatment rate *η* by 10% decreases (or increases) $R_{0}$ by 9.004%; similarly, increasing (or decreasing) the natural death in humans, *μ*_*H*_, by 10% increases (or decreases) $R_{0}$ by 5.710%. In the same way, increasing (or decreasing) the proportion of recruitment rate of humans, Λ_*H*_, by 10% increases (or decreases) $R_{0}$ by 5.5009%. Further, increasing (or decreasing) the rate of exposed humans moving into infectious class *χ*_*H*_, by 10% increases (or decreases) $R_{0}$ by 5.508%. In addition, increasing (or decreasing) the probability of mosquitoes getting infected, *β*_*H*_, by 10% increases (or decreases)$R_{0}$ by 4.630%. Wile increasing (or decreasing) the mosquitoes recruitment rate, Λ_*V*_ by 10% increases (or decreases) *R*_0_ by 4.490%. Furthermore, increasing (or decreasing) the rate flow from *E*_*v*_ to *I*_*v*_, *δ*_*V*_, by 10% increases (or decreases) $R_{0}$ by 2.641%. On the other hand, increasing (or decreasing) the human recovery rate due to treatment, *γ* by 10% increases (or decreases) $R_{0}$ by 1.809%

## 7 Analysis of optimal control

In this section, we make uses of Pontryagin’s Maximum Principle in order to come out with the necessary conditions that establishes the presence of optimal control of the Zika virus *SEIR* model. We include time dependent controls in the Zika *SEIR* model and endeavour to explore the appropriate optimal strategy for putting the Zika vius under control. We use three control variables, the control *u*_1_(*t*) represents the efforts on preventing zika infections through bednets, the control on treatment of zika infected individuals *u*_2_(*t*) and the third control *u*_3_(*t*) represents the efforts through insecticides spray against mosquito. In this regard, the following objective functional are taking into consideration our objective functional is similar to what is in the literature [9, 36, 37],

$$\begin{array}{c} J\left(u_{1},u_{2},u_{3}\right)=\int_{0}^{t_{f}}\left(BE_{H}+CI_{H}+DE_{V}+EI_{V}+\frac{a_{1}}{2}u_{1}^{2}+\frac{a_{2}}{2}u_{2}^{2}+\frac{a_{3}}{2}u_{3}^{2}\right)dt,. \end{array}$$ (18)

where *B*, *C*, *D*, *E* are the balancing cost factors due to scales and *a*_1_, *a*_2_ and *a*_3_ denote the weighting constants for making uses of bednets which has the potential of reducing the spread of the disease(prevention), effective treatment activities which include the efficacy of the drugs and encouraging patients to take their drugs timely and effective and availability of insecticide spraying against all stages of mosquitoes. The costs associated with prevention, treatment and insecticide are taken be of the form of nonlinear. Thus, we endeavour to anticipate an optimal control $u_{1}^{*},u_{2}^{*}$ and $u_{3}^{*}$ such that, $J\left(u_{1}^{*},\;u_{2}^{*},u_{3}^{*}\right)=\min J\left(u_{1},\;u_{2},u_{3}\right),\Gamma =\{\left(u_{1},\;u_{2},u_{3}\right)|0\leq u_{i}\leq 1,\;i=1,2,3\}.$

$$\begin{array}{c} \left\{ \begin{array}{c} \frac{d}{dt}S_{H}=\Lambda_{H}-\left(1-u_{1}\right)\beta_{H}S_{H}\left(I_{V}+\rho I_{H}\right)-\mu_{H}S_{H},\\ \frac{d}{dt}E_{H}=\left(1-u_{1}\right)\beta_{H}S_{H}\left(I_{V}+\rho I_{H}\right)-\left(\mu_{H}+\chi_{H}\right)E_{H},\\ \frac{d}{dt}I_{H}=\chi_{H}E_{H}-\left(\mu_{H}+u_{2}\gamma +\eta \right)I_{H},\\ \frac{d}{dt}R_{H}=u_{2}\gamma I_{H}-\mu_{H}R_{H},\\ \frac{d}{dt}S_{V}=\Lambda_{V}-\left(1-u_{1}\right)\beta_{V}S_{V}I_{H}-u_{3}\mu_{V}S_{V},\\ \frac{d}{dt}E_{V}=\left(1-u_{1}\right)\beta_{V}S_{V}I_{H}-\left(\delta_{V}+u_{3}\mu_{V}\right)E_{V},\\ \frac{d}{dt}I_{V}=\delta_{V}E_{V}-u_{3}\mu_{V}I_{V}. \end{array}\right. \end{array}$$ (19)

The necessary conditions that an optimal solution must conform is emanated from the Pontryagin Maximum Principle [38]. This concept translates Eqs (18) and (19) into a kind of problem characterised with minimizing pointwise a Hamiltonian *H*, with respect to *u*_1_, *u*_2_ and *u*_3_

$$\begin{array}{c} \begin{array}{c} H=BE_{H}+CI_{H}+DE_{V}+EI_{V}+a_{1}\mu_{1}^{2}+a_{2}\mu_{2}^{2}+a_{3}\mu_{3}^{2}\\ +\lambda_{S_{H}}\left\{\Lambda_{H}-\left(1-\mu_{1}\right)\beta_{H}S_{H}\left(I_{V}+\rho I_{H}\right)-\mu_{H}S_{H}\right\}\\ +\lambda_{E_{H}}\left\{\left(1-\mu_{1}\right)\beta_{H}S_{H}\left(I_{V}+\rho I_{H}\right)-\left(\mu_{H}+\chi_{H}\right)E_{H}\right\}\\ +\lambda_{I_{H}}\left\{\chi_{H}E_{H}-\left(\mu_{H}+u_{2}\gamma +\eta \right)I_{H}\right\}\\ +\lambda_{R_{H}}\left\{u_{2}\gamma I_{H}-\mu_{H}R_{H},\right\}\\ +\lambda_{S_{V}}\left\{\Lambda_{V}-\left(1-\mu_{1}\right)\beta_{V}S_{V}I_{H}-\mu_{V}u_{3}S_{V}\right\}\\ +\lambda_{E_{H}}\left\{\left(1-\mu_{1}\right)\beta_{V}S_{V}I_{H}-\left(\delta_{V}+u_{3}\mu_{V}\right)E_{V}\right\}\\ +\lambda_{I_{V}}\left\{\delta_{V}E_{V}-\mu_{V}u_{3}I_{V}\right\} \end{array} \end{array}$$ (20)

where *λ*_*S*_*H*__, *λ*_*E*_*H*__, *λ*_*I*_*H*__, *λ*_*R*_*H*__, *λ*_*S*_*V*__, *λ*_*E*_*V*__ and *λ*_*I*_*V*__ constitute the adjoint variables or co-state variables. The system solution is attained by appropriately taking partial derivatives of the Hamiltonian Eq (20) with respect to the associated state variable.

**Theorem 7.1**. *Given optimal controls* $u_{1}^{*},u_{2}^{*},u_{3}^{*}$ *and solutions* *S*_*H*_, *E*_*H*_, *I*_*H*_, *R*_*H*_, *S*_*V*_, *E*_*V*_, *I*_*V*_ *of the corresponding state System* (18) *and* (19) *that minimize* *J*(*u*_1_, *u*_2_, *u*_3_) *over* *Γ*. *Then there exists adjoint variables* *λ*_*S*_*H*__, *λ*_*E*_*H*__, *λ*_*I*_*H*__, *λ*_*R*_*H*__, *λ*_*S*_*V*__, *λ*_*E*_*V*__, *λ*_*I*_*V*__ *satisfying*

$$\begin{array}{c} \frac{-d\lambda_{i}}{dt}=\frac{\partial H}{\partial i} \end{array}$$ (21)

where *i* = *S*_*H*_, *E*_*H*_, *R*_*H*_, *I*_*V*_, *S*_*V*_, *E*_*V*_, *I*_*V*_ and with transversality conditions

$$\begin{array}{c} \lambda_{S_{H}}\left(t_{f}\right)=\lambda_{E_{H}}\left(t_{f}\right)=\lambda_{I_{H}}\left(t_{f}\right)=\lambda_{R_{H}}\left(t_{f}\right)=\lambda_{S_{V}}\left(t_{f}\right)=\lambda_{E_{V}}\left(t_{f}\right)=\lambda_{I_{V}}\left(t_{f}\right)=0 \end{array}$$ (22)

and

$$\begin{array}{c} u_{1}^{*}=\min\left\{1,\max(0,\frac{S_{H}\beta_{H}I_{V}\left(\lambda_{E_{H}}-\lambda_{S_{H}}\right)+S_{V}\beta_{V}I_{H}\left(\lambda_{E_{V}}-\lambda_{S_{V}}\right)}{a_{1}})\right\}, \end{array}$$ (23)

$$\begin{array}{c} u_{2}^{*}=\min\left\{1,\max(0,\frac{\gamma I_{H}\left(\lambda_{I_{H}}-\lambda_{R_{H}}\right)}{a_{2}})\right\}, \end{array}$$ (24)

$$\begin{array}{c} u_{3}^{*}=\min\left\{1,\max(0,\frac{\mu_{V}S_{V}\lambda_{S_{V}}+\mu_{V}E_{V}\lambda_{E_{V}}+\mu_{V}I_{V}\lambda_{I_{V}}}{a_{3}})\right\}. \end{array}$$ (25)

**Proof:** Corollary 4.1 of Fleming and Rishel [39] provides the condition of possible existence of an optimal control based on the convexity of the integrand of *J* with respect to *u*_1_, *u*_2_ and *u*_3_, a *priori* boundedness of the state solutions, and the *Lipschitz* characteristics of the state system in line with the state variables. The Hamiltonian function determines at the optimal control level leads to the governing adjoint variables. Thus, the adjoint equations can be rearranged as

$$\begin{array}{c} \begin{array}{c} -\frac{d\lambda_{S_{H}}}{dt}=\mu_{H}\lambda_{S_{H}}+\left(1-u_{1}\right)\beta_{H}\left(\lambda_{S_{H}}-\lambda_{E_{H}}\right)\left(I_{V}+\rho I_{H}\right)\\ -\frac{d\lambda_{E_{H}}}{dt}=-B+\left(\mu_{H}+\chi_{H}\right)\lambda_{E_{H}}-\chi_{H}\lambda_{I_{H}}\\ -\frac{d\lambda_{I_{H}}}{dt}=-C+\left(\mu_{H}+u_{2}\gamma +\eta \right)\lambda_{I_{H}}-\left(u_{2}\gamma \right)\lambda_{R_{H}}+\rho \left(1-u_{1}\right)\beta_{H}S_{H}\left(\lambda_{S_{H}}-\lambda_{E_{H}}\right)\\ +\left(1-u_{1}\right)\beta_{V}S_{V}\left(\lambda_{S_{V}}-\lambda_{E_{V}}\right)\\ -\frac{d\lambda_{R_{H}}}{dt}=\mu_{H}\lambda_{R_{H}}\\ -\frac{d\lambda_{S_{V}}}{dt}=\left(1-u_{1}\right)\beta_{V}I_{H}\left(\lambda_{S_{V}}-\lambda_{E_{V}}\right)+\left(\mu_{V}u_{3}\right)\lambda_{S_{V}}\\ -\frac{d\lambda_{E_{V}}}{dt}=-D+-\left(\delta_{V}+u_{3}\mu_{V}\right)\lambda_{E_{V}}-\delta_{V}\lambda_{I_{V}}\\ -\frac{d\lambda_{I_{V}}}{dt}=-E+\left(1-u_{1}\right)\beta_{H}S_{H}\left(\lambda_{S_{H}}-\lambda_{E_{H}}\right)+\mu_{V}u_{3}\lambda_{I_{V}} \end{array} \end{array}$$ (26)

## 8 Numerical simulations

In this section, we present numerical simulation solutions as illustration which is obtained using MATLAB program. The Table 2 presents the parameter values used for the simulations.

**Table 2:** Description of variables and parameters of the model.

Parameter	Description	value	Ref
*β*_*H*_	Probability of humans getting infected	0.2 *day*^−1^	[40]
*β*_*V*_	Probability of mosquitoes getting infected	0.09	[3]
*μ*_*H*_	Natural death rate in humans	1/(365x60) *day*^−1^	[3]
*μ*_*V*_	Natural death rate in mosquitoes	1/14	[9]
*χ*_*H*_	The rate of exposed humans moving into infectious class	0.01	[40]
Λ_*H*_	Recruitment rate of humans	100 *day*^−1^	assumed
Λ_*V*_	Mosquito recruitment rate	1000 *day*^−1^	assumed
*γ*	Human recovery rate due to treatment	1000 *day*^−1^	assumed
*ρ*	Human factor transmission rate	0.05 *day*^−1^	assumed
*η*	Human infected treatment rate	0.2 *day*^−1^	[9]
*δ*_*V*_	The rate flow from *E*_*V*_ to *I*_*V*_	0.05 *day*^−1^	assumed

### 8.1 Prevention (*u*_1_) and treatment (*u*_2_) control only

**Fig 2:**
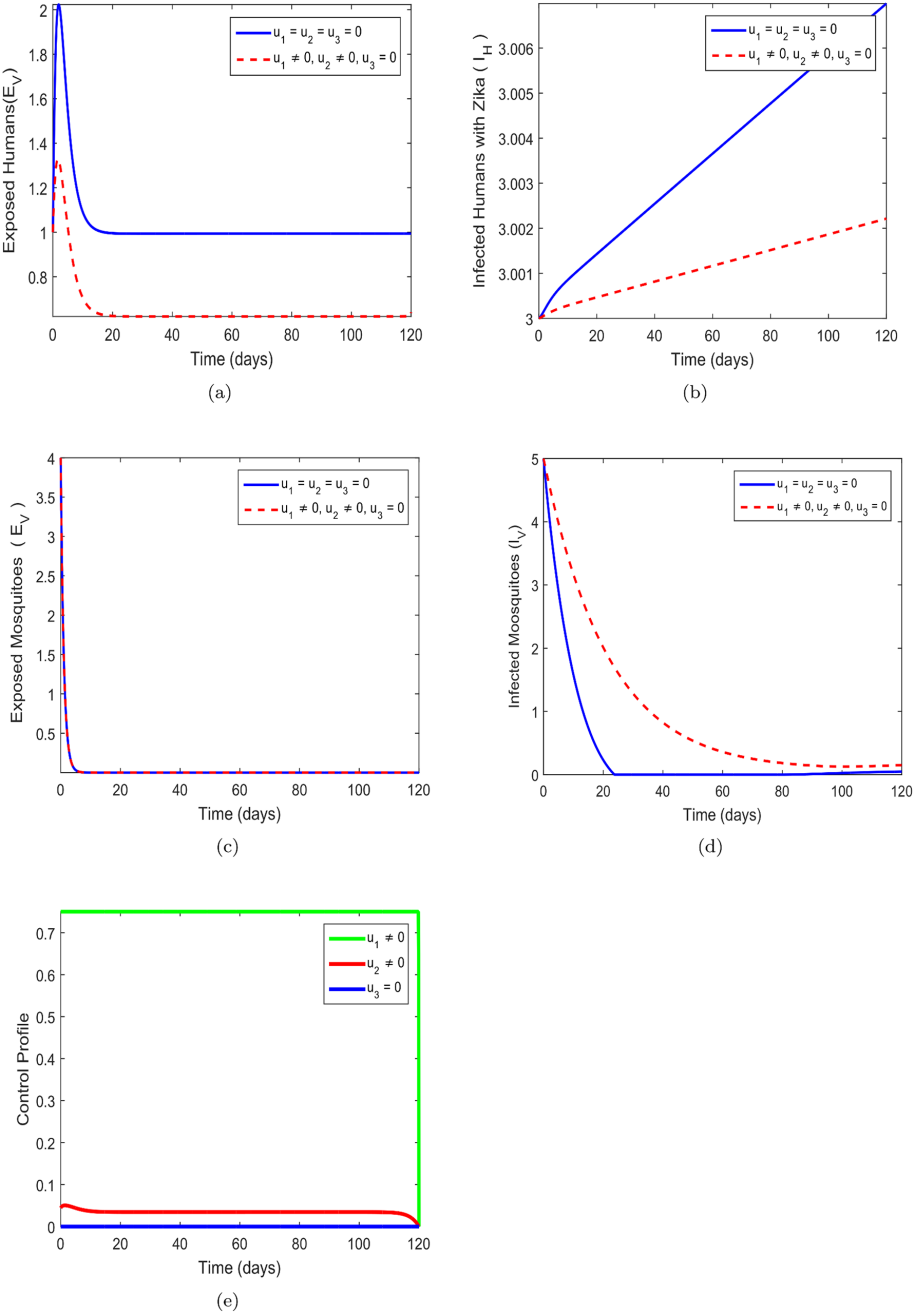
Simulations of the model showing the effect of Zika prevention and treatment only on transmission. Fig 2(a)-(e) respectively represent the behavior of exposed human, infected human, exposed mosquitos, infected mosquitos and control profile. *u*_1_ = *u*_2_ = *u*_3_ = 0 represents system without control while *u*_1_ = *u*_2_ ≠ 0, *u*_3_ = 0, shows control system. Fig 2(a) *u*_1_ = *u*_2_ = *u*_3_ = 0-without control system and *u*_1_ = *u*_2_ ≠ 0, *u*_3_ = 0 control system, Fig 2(b) *u*_1_ = *u*_2_ = *u*_3_ = 0-without control system and *u*_1_ = *u*_2_ ≠ 0, *u*_3_ = 0 control system, Fig 2(c) *u*_1_ = *u*_2_ = *u*_3_ = 0-without control system and *u*_1_ = *u*_2_ ≠ 0, *u*_3_ = 0 control system, Fig 2(d) *u*_1_ = *u*_2_ = *u*_3_ = 0-without control system and *u*_1_ = *u*_2_ ≠ 0, *u*_3_ = 0 control system, Fig 2(e) *u*_1_ = *u*_2_ = *u*_3_ = 0-without control system and *u*_1_ = *u*_2_ ≠ 0, *u*_3_ = 0 control system.

In this strategy, prevention measure of providing bednets *u*_1_ and the treatment efforts *u*_2_ are employed to optimize the objective function *J*, and at same time the insecticide spray control (*u*_3_) is set to zero. It is obvious in Fig 2(a) that there is a substantial difference between the number of exposed individuals *E*_*H*_ under control, compare to cases without control. Without the presence of the two controls the number of exposed humans appear to be increasing. The result depicted in Fig 2(b) clearly shows that the control strategy activated is effective to reduce the infected humans *I*_*H*_ under control, as not the case without control. The number of infected humans increases without the control strategies. Fig 2(c) showed that there is no significant different between the presence of control and without control in the exposed Mosquitoes. The obvious pattern is observed in Fig 2(d) that without control the infected mosquitoes are reducing than the presence of control. Fig 2(e) shows that the Zika prevention control *u*_1_ should be maintained at a maximum effort in the entire duration of the intervention at the same time control *u*_2_ which deals with prevention should be kept about 6% during the 120 days.

### 8.2 Prevention (*u*_1_) and insecticide control (*u*_3_) only

**Fig 3:**
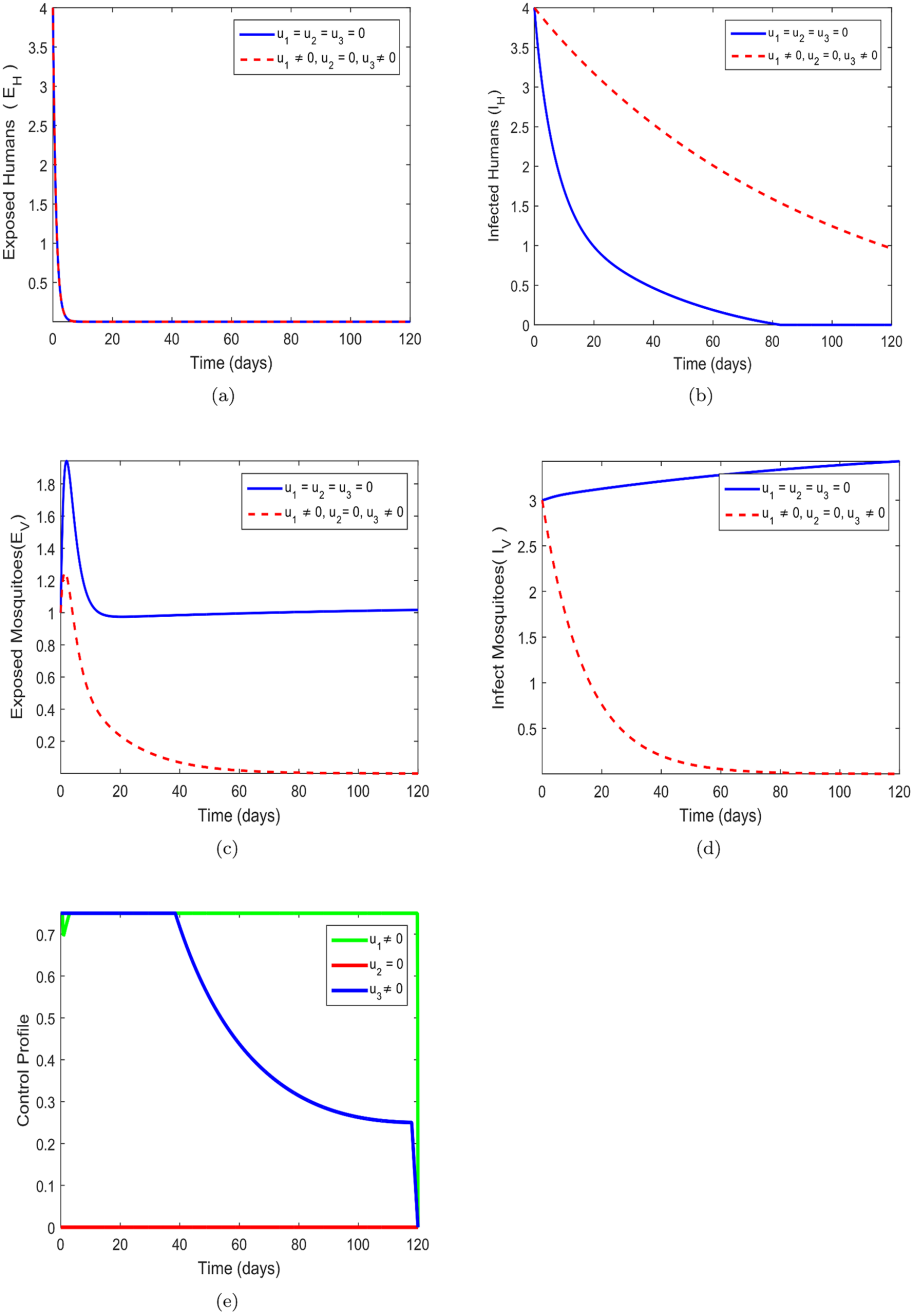
Simulations of the model showing the effect of Zika prevention and treatment only on transmission. Fig 3(a)-(e) respectively represent the behavior of exposed human, infected human, exposed mosquitos, infected mosquitos and control profile. *u*_1_ = *u*_2_ = *u*_3_ = 0 represents system without control while *u*_1_ = *u*_3_ ≠ 0, *u*_2_ = 0, shows control system. Fig 3(a) *u*_1_ = *u*_2_ = *u*_3_ = 0-without control system and *u*_1_ = *u*_3_ ≠ 0, *u*_2_ = 0 control system, Fig 3(b) *u*_1_ = *u*_2_ = *u*_3_ = 0-without control system and *u*_1_ = *u*_3_ ≠ 0, *u*_2_ = 0 control system, Fig 3(c) *u*_1_ = *u*_2_ = *u*_3_ = 0-without control system and *u*_1_ = *u*_3_ ≠ 0, *u*_2_ = 0 control system, Fig 3(d) *u*_1_ = *u*_2_ = *u*_3_ = 0-without control system and *u*_1_ = *u*_3_ ≠ 0, *u*_2_ = 0 control system, Fig 3(e) *u*_1_ = *u*_2_ = *u*_3_ = 0-without control system and *u*_1_ = *u*_3_ ≠ 0, *u*_2_ = 0 control system.

In this strategy, prevention effort targeting at making effective uses of bednets *u*_1_ and the insecticide spray control (*u*_3_) are explored while control *u*_2_ is set to zero is employed to optimized the objective function *J*. We can infer from Fig 3(a) that there is no difference between the presence of the two controls activated and without the controls in the number of exposed humans *E*_*H*_ bednet *u*_1_. In Fig 3(b) the number of infected humans is increased despite the activation of controls *u*_1_ and *u*_2_ and this indicated that just provision of bednet and treatment of humans would not reduce the spread of Zika virus. The control strategy in Fig 3(c) is effective as there is a substantial difference between the presence of control and without control. in the number of exposed mosquitoes *E*_*V*_. It can be infer that if effective mechanisms of praying mosquitoes are put in place little attention can be put in the provision of bednets in humans. significant difference is also shown in Fig 3(b) which suggest that this control strategy. There is a significant difference between the presence of the two controls and without the control as seen in Fig 3(d). The use of bednets and insecticide pray reduces the number of infected mosquitoes *I*_*V*_ and would eventually reduce the spread of the disease. The control profile in Fig 3(e) stipulates that this strategy would require that control *u*_1_ must be maintained for a maximum effort of 120 days throughout the process while control *u*_3_ must be kept constant for maximum effort 100% for 40 days and gradually reduce to 22% within the rest of the 120 days. The coreol *u*_2_ is kept zero throughout the process.

### 8.3 Treatment (*u*_2_) and insecticide (*u*_3_) only

**Fig 4:**
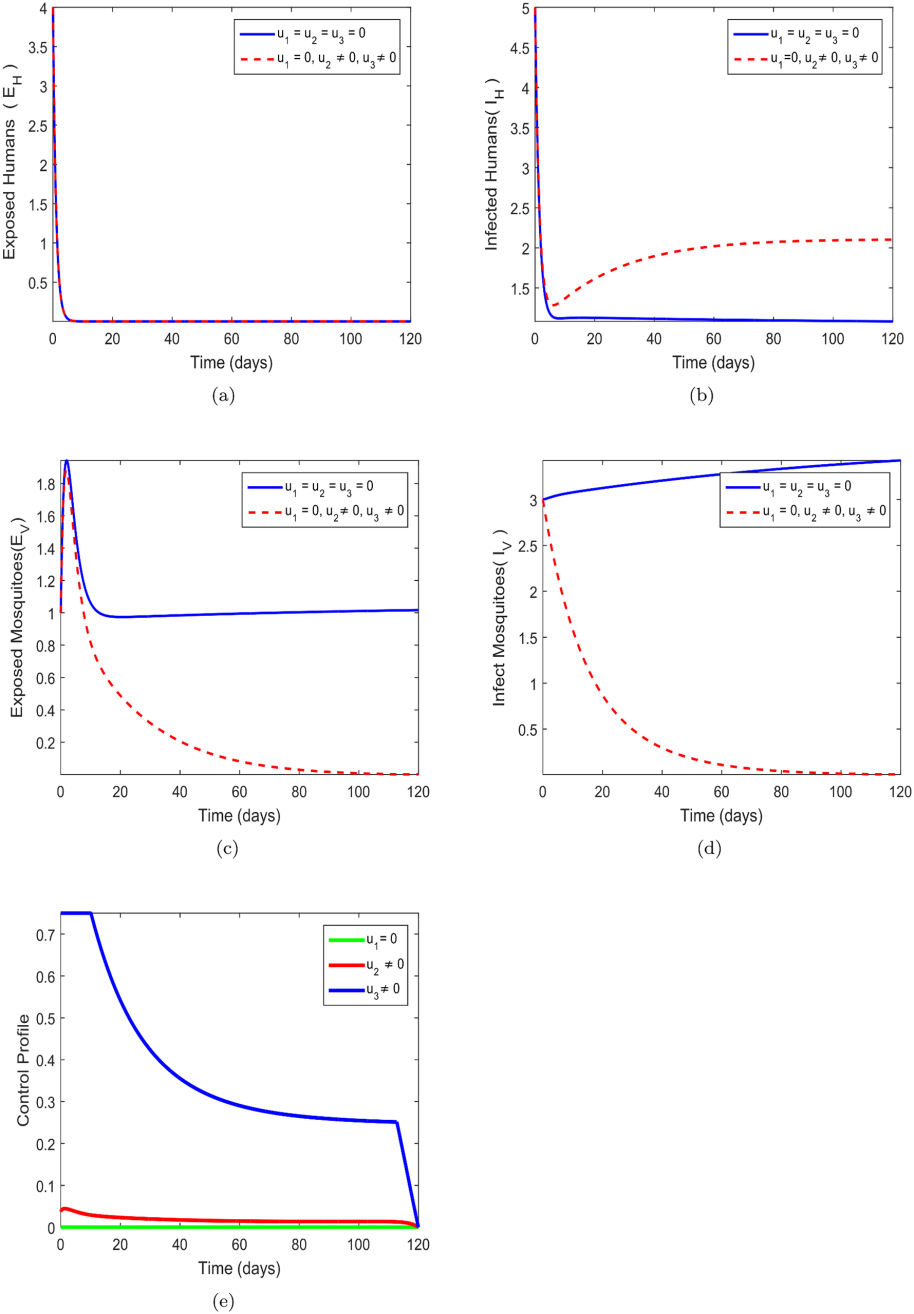
Simulations of the model showing the effect of Zika prevention and treatment only on transmission. Fig 4(a)-(e) respectively represent the behavior of exposed human, infected human, exposed mosquitos, infected mosquitos and control profile. *u*_1_ = *u*_2_ = *u*_3_ = 0 represents system without control while *u*_2_ = *u*_3_ ≠ 0, *u*_1_ = 0, shows control system. Fig 4(a) *u*_1_ = *u*_2_ = *u*_3_ = 0-without control system and *u*_2_ = *u*_3_ ≠ 0, *u*_1_ = 0 control system, Fig 4(b) *u*_1_ = *u*_2_ = *u*_3_ = 0-without control system and *u*_2_ = *u*_3_ ≠ 0, *u*_1_ = 0 control system, Fig 4(c) *u*_1_ = *u*_2_ = *u*_3_ = 0-without control system and *u*_2_ = *u*_3_ ≠ 0, *u*_1_ = 0 control system, Fig 4(d) *u*_1_ = *u*_2_ = *u*_3_ = 0-without control system and *u*_2_ = *u*_3_ ≠ 0, *u*_1_ = 0 control system, Fig 4(e) *u*_1_ = *u*_2_ = *u*_3_ = 0-without control system and *u*_2_ = *u*_3_ ≠ 0, *u*_1_ = 0 control system.

In this strategy, treatment efforts *u*_2_ and the insecticide spray control (*u*_3_) are employed to optimize the objective function *J*, at the same time the prevention control *u*_1_ is set to zero. We can infer from Fig 4(a) that there no significant difference between the number of exposed humans *E*_*H*_ in the presence of control strategy and without control strategy. This indicates that the strategy is not the best way to reduce the number of humans getting exposed to the zika virus disease. The result in Fig 4(b) shows that the control strategy is not the best way to reduce the number of infected humans. The control strategy is more effective in Fig 4(c) as the number of exposed mosquitoes *E*_*V*_ are substantially minimized. In fact, without the presence of control in Fig 4(c) the number of exposed mosquitoes *E*_*V*_ is increasing. It is clear that in Fig 4(d) there is a vast different between the number of infected mosquitoes *I*_*V*_ the presence of control and without control strategy. There presence of control strategy is capable of minimizing the number of infected mosquitoes *I*_*V*_ in the communities. The control profile in Fig 4(c) indicates that the Zika prevention control *u*_3_ should be maintained at maximum effort 100% for 16 days and finally decreasing till the end of 120 days while control *u*_2_ must be kept at a maximum effort 5% for 5 days and immediately decreasing zero in the entire 120 days. intervention.

### 8.4 Prevention, treatment and insecticide (*u*_1_, *u*_2_, *u*_3_)

**Fig 5:**
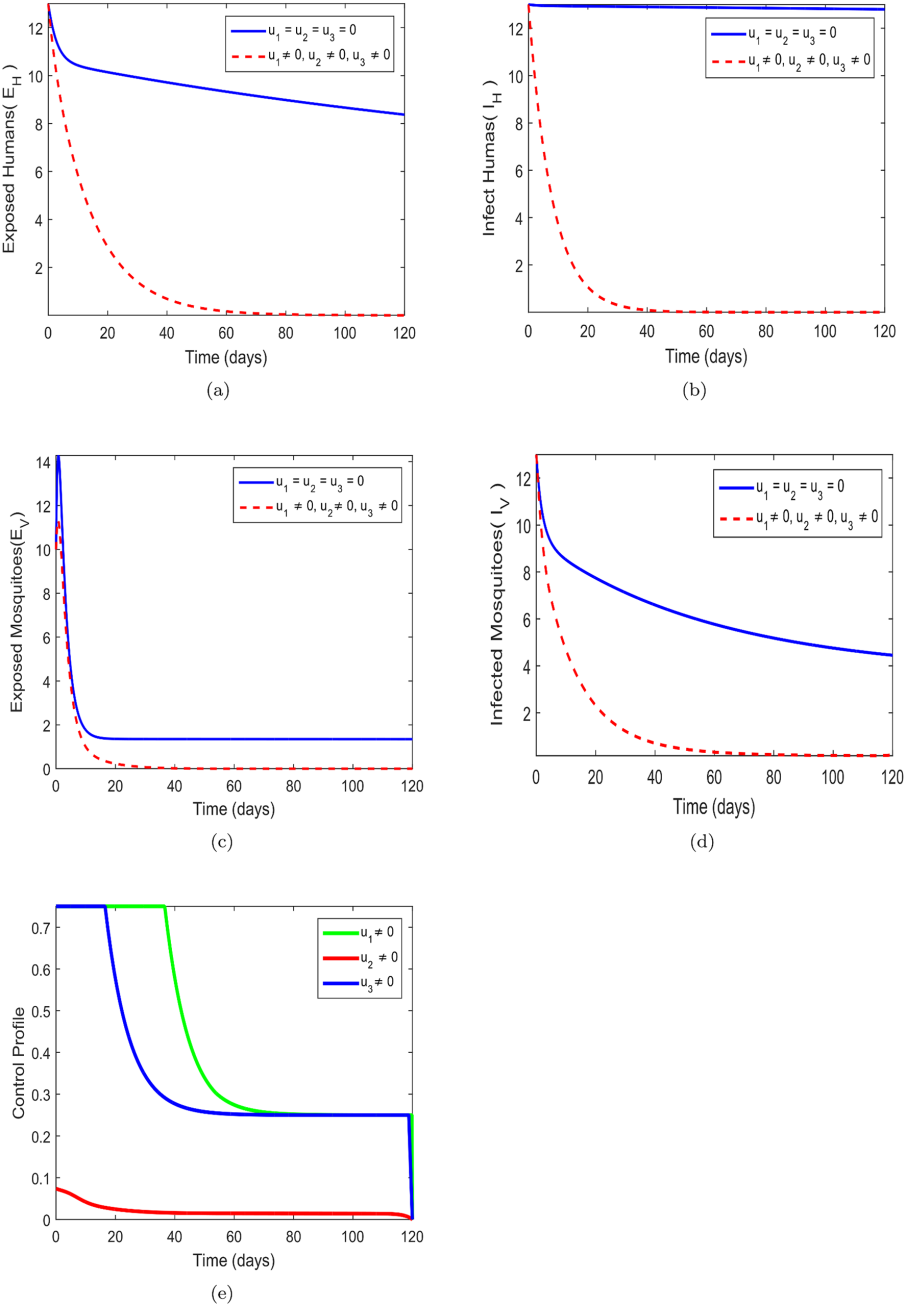
Simulations of the model showing the effect of Zika prevention and treatment only on transmission. Fig 5(a)-(e) respectively represent the behavior of exposed human, infected human, exposed mosquitos, infected mosquitos and control profile. *u*_1_ = *u*_2_ = *u*_3_ = 0 represents system without control while *u*_1_ = *u*_2_ = *u*_3_ ≠ 0, shows control system. Fig 5(a) *u*_1_ = *u*_2_ = *u*_3_ = 0-without control system and *u*_1_ = *u*_2_ = *u*_3_ ≠ 0, control system, Fig 5(b) *u*_1_ = *u*_2_ = *u*_3_ = 0-without control system and *u*_1_ = *u*_2_ = *u*_3_ ≠ 0 control system, Fig 5(c) *u*_1_ = *u*_2_ = *u*_3_ = 0-without control system and *u*_1_ = *u*_2_ = *u*_3_ ≠ 0 control system, Fig 5(d) *u*_1_ = *u*_2_ = *u*_3_ = 0-without control system and *u*_1_ = *u*_2_ = *u*_3_ ≠ 0 control system, Fig 5(e) *u*_1_ = *u*_2_ = *u*_3_ = 0-without control system and *u*_1_ = *u*_2_ = *u*_3_ ≠ 0 control system.

In this strategy, all the three controls are explored in order to optimize (*u*_1_, *u*_2_, *u*_3_). It obvious in Fig 5(a) that a vast significant difference between number of exposed humans *E*_*H*_ in the presence of control and without control. The activation of all the controls has the a greater effect of minimizing the number of exposed humans in the communities. The in Fig 5(b) further indicates that there substantial difference between the presence of control and without control. This shows that the control mechanisms are able to reduce the number of infected humans *I*_*H*_ within the communities. In the absence control the the infection will be spreading at a faster rate. There is relatively significant difference between the presence of control and without control as in Fig 5(c). In fact communities where zika virus are presence should be to take precaution measures to avoid exposure to the infected virus.Fig 5(d) suggest that the there is substantial different between the the presence of control and without control. This further suggests that the application of all the three control is the best strategy to minimize the number of infected mosquitoes *V*_*I*_ which will eventually can lead to the reduction of the spread of zika virus. The control profile in Fig 5(c) suggests that control *u*_1_ ought to be kept at a maximum 100% for about 40 days and gradually reduce to 25% and kept same within the entire 120 days period. The control *u*_2_ is just maintain at 8% and then gradually decrease and maintain in the entire 120 days. The control *u*_3_ is kept at a maximum 100% for 20 days then decrease to 25% which is maintain throughout the entire 120 days.

## 9 Conclusion

In this work, we studied a deterministic Zika virus model. The basic properties of the proposed model is investigated in addition to the basic reproduction $R_{0}$ without control. The steady states of the model is studied and both disease free and endemic equilibrium is locally asymptotically stable. The disease free equilibrium is found to be globally asymptotically is stable. The central manifold theory is employed to study the stability of endemic equilibrium and also found to be asymptotically stable. Optimal time control is incorporated into the proposed model namely bednets, treatment and spraying of insecticide. The Pontryagin’s Maximum Principle is explored and used to determine the essential conditions usually necessary for effective control of zika virus. The numerical simulation results obtained suggest that the best strategy to minimize the the spread of zika virus is to optimize all the three controls. The reduction of the disease can only be attained when needed attention of all the thee controls are taken into account. The results presented are clear and the public health implications are provided.
